# MEG Sensor Selection for Neural Speech Decoding

**DOI:** 10.1109/access.2020.3028831

**Published:** 2020-10-06

**Authors:** DEBADATTA DASH, ALAN WISLER, PAUL FERRARI, ELIZABETH MOODY DAVENPORT, JOSEPH MALDJIAN, JUN WANG

**Affiliations:** 1 Department of Electrical and Computer Engineering, The University of Texas at Austin, Austin, TX 78712, USA; 2 Department of Neurology, Dell Medical School, The University of Texas at Austin, Austin, TX 78712, USA; 3 Department of Speech, Language, and Hearing Sciences, University of Texas at Austin, Austin, TX 78712, USA; 4 MEG Laboratory, Dell Children’s Medical Center, Austin, TX 78723, USA; 5 Department of Psychology, The University of Texas at Austin, Austin, TX 78712, USA; 6 Department of Radiology, University of Texas at Southwestern, Dallas, TX 75390, USA

**Keywords:** Autoencoder, brain-computer interface, forward selection algorithm, magnetoencephalography, neural speech decoding, OPM, SVM

## Abstract

Direct decoding of speech from the brain is a faster alternative to current electroencephalography (EEG) speller-based brain-computer interfaces (BCI) in providing communication assistance to locked-in patients. Magnetoencephalography (MEG) has recently shown great potential as a non-invasive neuroimaging modality for neural speech decoding, owing in part to its spatial selectivity over other high-temporal resolution devices. Standard MEG systems have a large number of cryogenically cooled channels/sensors (200 – 300) encapsulated within a fixed liquid helium dewar, precluding their use as wearable BCI devices. Fortunately, recently developed optically pumped magnetometers (OPM) do not require cryogens, and have the potential to be wearable and movable making them more suitable for BCI applications. This design is also modular allowing for customized montages to include only the sensors necessary for a particular task. As the number of sensors bears a heavy influence on the cost, size, and weight of MEG systems, minimizing the number of sensors is critical for designing practical MEG-based BCIs in the future. In this study, we sought to identify an optimal set of MEG channels to decode imagined and spoken phrases from the MEG signals. Using a forward selection algorithm with a support vector machine classifier we found that nine optimally located MEG gradiometers provided higher decoding accuracy compared to using all channels. Additionally, the forward selection algorithm achieved similar performance to dimensionality reduction using a stacked-sparse-autoencoder. Analysis of spatial dynamics of speech decoding suggested that both left and right hemisphere sensors contribute to speech decoding. Sensors approximately located near Broca’s area were found to be commonly contributing among the higher-ranked sensors across all subjects.

## I. INTRODUCTION

Speech processing involves a complex yet hierarchical execution of oromotor tasks underpinned by continuous cross-talk among various cortical areas. This process can be impaired with neurodegenerative diseases such as amyotrophic lateral sclerosis (ALS), a motor neuron disease that causes progressive motor paralysis. Late-stage ALS can lead to a state of complete paralysis but aware, called locked-in syndrome [1], which impairs motor functions including speech production. Despite its devastating effects on motor control, ALS spares cognitive processes [2], leaving the brain to be the only viable source of communication for these patients. Current commercially available brain-computer interfaces (BCI) use electroencephalography (EEG) recordings of visual and attention correlates to spell characters from a screen, providing a means of communication for these patients [3]–[5]. However, the slow communication rate of these BCI spellers (under 10 words per minute) is a major obstacle for users [6]–[8] as they may experience fatigue. Acknowledging this limitation, recent studies have attempted on direct neural speech decoding, which involves designing classifiers corresponding to different speech units [9]–[14] or direct mapping of brain to text/speech [15]–[18]. These speech decoding driven BCIs or speech-BCIs have the potential of offering real-time communication assistance to locked-in patients [14], [18]–[20].

To develop a speech-BCI, the choice of neuroimaging modality is of paramount importance. Cortical speech processing is dynamic and fast, involving multiple brain regions undergoing parallel processes. Hence, to effectively characterize speech information, the modality must have good enough spatial resolution to localize focal sources and a temporal resolution that can capture the rapid dynamics of speech-neural processing. Extensive decoding research has been carried out with EEG, due in large part to its low cost, non-invasiveness, and easy data acquisition capability. Classification of individualized speech units, such as imagined syllables/phonemes/digits or even words have been thoroughly investigated with EEG [21]–[26] providing strong evidence for speech decoding using non-invasive signals. However, there are several drawbacks with this modality such as signal distortion by neural tissue boundaries during recording and reference dependence, which degrade the decoding performance.

Electrocorticography (ECoG) has also been explored for speech decoding, where researchers have successfully synthesized speech directly from neural signals [16]–[18]. ECoG has been shown to be effective for closed-set classification-based speech decoding [27]–[29] as well as open-set recognition of phonemes [15] or characters [30]. Despite this success, ECoG is invasive, requiring a craniotomy and surgery to implant electrodes into patients’ brains. Additionally, it may be challenging to establish long-term biocompatibility between the ECoG electrode grid and the brain. Although some recent ECoG BCI studies have shown promising results for ALS patients with relatively longer-term motor applications via a few (2 – 4) electrodes those were placed around the motor cortex [31]–[33], it is unknown how many electrodes are needed for speech production decoding. Moreover, prior ECoG studies have analyzed only part of the brain, as ECoG electrodes are typically implanted only into the areas prone to epileptic seizures. Hence, ECoG lacks a full-scale utilization of brain function for speech processing.

Magnetoencephalography (MEG) is a non-invasive, whole-head neuroimaging modality well suited for neural speech decoding. This functional neuroimaging technique measures patterns of magnetic fields produced by the small cortical neuronal currents using highly sensitive magnetometers and gradiometers. Although the extracranially measured magnetic fields by MEG can be influenced by conductivity boundaries, the effects can be corrected using various modeling strategies [34]. It has a high spatial resolution (3 – 10mm) as well as an excellent temporal resolution (*<* 1ms). This combination of high spatial and temporal resolution makes MEG an excellent neurophysiological measurement modality to image complex brain activity during speech production. MEG has been previously used to investigate numerous mechanisms underlying speech, including auditory cortical responses to speech perception [35]–[37], neural phase-locking patterns via discriminating speech stimuli [38], and cortical network oscillations for speech production mechanisms [39]. These studies provide strong support for the efficacy of using MEG for speech-based BCI studies. Specifically, the advantages in the high temporal resolution of MEG has been proven to be effective by several MEG studies including the superiority of MEG recordings for speech production [40], neural tracking of speech envelope [41], investigating temporal dynamics of communicative language processing (naming and requesting) [42], temporal patterns of neural activations in speech [43], automatic neural speech activity recognition [44], and real-time voice activity detection from neuromagnetic signals (NeuroVAD) [45]. These studies provide strong evidence for the ability of MEG to decode speech from neuromagnetic signals. Moreover, our prior works [10]–[12], [20], [46], have demonstrated the effectiveness of neuromagnetic signals for high accuracy speech decoding.

Despite the advantages of neuromagnetic signals, there remain technological barriers to the widespread implementation of MEG machines in speech decoding applications. Current MEG measurements are based on SQUID (superconducting quantum interference device) arrays housed within a liquid helium dewar for cryogenic cooling with a vacuum gap separating sensors and scalp. This arrangement makes the MEG machine bulky, non-portable, and very costly. The development of optically pumped magnetometers (OPM) [47]–[52] can overcome many of these limitations. OPMs leverage the quantum mechanical properties of alkali atoms to measure small magnetic fields in the brain [53] in contrast to the SQUID-MEG, which uses magnetic coils. Nevertheless, both measure the same neuromagnetic activity in the brain. OPM-MEG systems are relatively light, can operate at room temperature, potentially portable, and individual sensors can be slotted onto a 3D printed scanner cast form-fitted to the individuals’ head. The OPM-MEG (on-scalp-MEG) system improves sensor-to-cortex proximity compared to the conventional MEG (in-helmet-MEG) systems thereby providing improved spatial resolution, higher signal magnitude, and better separation of source activations [54], [55]. These advances have made OPM-based MEG systems far more suitable as a wearable device. Another major advantage of this next-generation MEG system is the option of a modular design, in contrast to the present ‘one helmet fits all sensors’ MEG system. This opens up new possibilities for selecting sensor locations for studying specific neuro-cognitive operations including speech production. An optimal sensor set may prove instrumental for designing efficient and low-cost devices in the future. While OPMs currently lack the ability to operate outside of a shielded room, future hardware and software development may allow for a truly mobile device for patients. Although our current study used data collected using the traditional SQUID MEG, the feature extraction strategies and decoding algorithms would be the same if applied to data collected using OPM.

In the current study, we investigated the efficacy of using optimal sets of MEG sensors for neural speech decoding. We hypothesize that a set of optimally located sensors can be empirically chosen. A forward selection-based feature selection algorithm was used to classify neuromagnetic signals corresponding to different imagined and spoken phrases with superior performance compared to using the entire MEG sensor array. A feature selection approach selects the most significant subset from the whole feature set by removing irrelevant or redundant features from the data and thereby improves model performance, reduces computation, and facilitates a better understanding of the model learning. There are a number of intelligent feature selection or reduction approaches that have been proposed in literature including filter methods, wrapper methods, forward selection, backward selection, embedded methods or regularization, LASSO regression, Ridge regression, matrix factorization methods such as principal component analysis (PCA), independent component analysis (ICA), etc. [56]. In most of the cases, features are transformed into a new low-dimensional feature space. The objective of this study is to reduce the number of sensors in the sensor space itself such that locations of those optimal sensor set can be selected. The algorithm which we experimented for our objective was the forward selection algorithm [57], because of its inherent nature of iterative approach starting from the smallest dimension to the optimal set leading to faster computation. We chose a support vector machine (SVM) decoder for classification considering its faster training ability with small datasets and well suitability when combined with feature selection algorithms [58]. Moreover, SVMs have been proven to be effective in various classification tasks including our previous works with MEG [45], [59]. In addition, we also investigated a stacked autoencoder-based dimension reduction strategy to reconfirm the improved decoding accuracy. We chose autoencoder for the dimension reduction task considering its effectiveness and previous successful MEG studies [60], [61]. We implemented both forward selection-based feature selection and autoencoder-based dimension reduction algorithms with an SVM decoder in a subject-dependent speech decoding perspective.

The major contributions of this study are as follows:

- This study presented a simple yet effective method for determining an optimally arranged sensor array that provides improved neural speech decoding of imagined and spoken phrases over the full sensor array. To our knowledge, this is the first MEG sensor selection for neural speech decoding work.
- This study also investigated a neural dimension reduction strategy using autoencoders to provide further evidence on the efficacy of reduced feature dimension-based speech decoding.
- Through analysis of the optimal arrays, we showed that spatial information underlying speech-related decoding can be revealed and that both hemispheres contribute to decoding, with the left hemisphere sensors being the most common across subjects despite tremendous individual differences in spatial distribution.

## II. MEG METHODS

### A. SUBJECTS

Seven healthy subjects without any history of neurological or developmental disorder (mean age: 41 ± 14 years; 3 females) participated in this study. All subjects were English speakers and right-handed. Informed consent was obtained from each subject prior to data acquisition, in compliance with the institutional review boards (IRB) of the participating institutions.

### B. STIMULI

Stimuli consisted of five commonly used short phrases borrowed from the list used in alternative and augmented communication: *1. Do you understand me; 2. That’s perfect; 3. How are you; 4. Good-bye; 5. I need help*. These were used as visual stimuli, displayed on a screen, one at a time, written in English. A stimulus dedicated computer running the STIM2 software (Compumedics, LTD) connected to a high-quality DLP projector was used to display the stimuli onto a back-projection screen situated at 90cm from the subjects.

### C. DATA ACQUISITION

**FIGURE 1.**
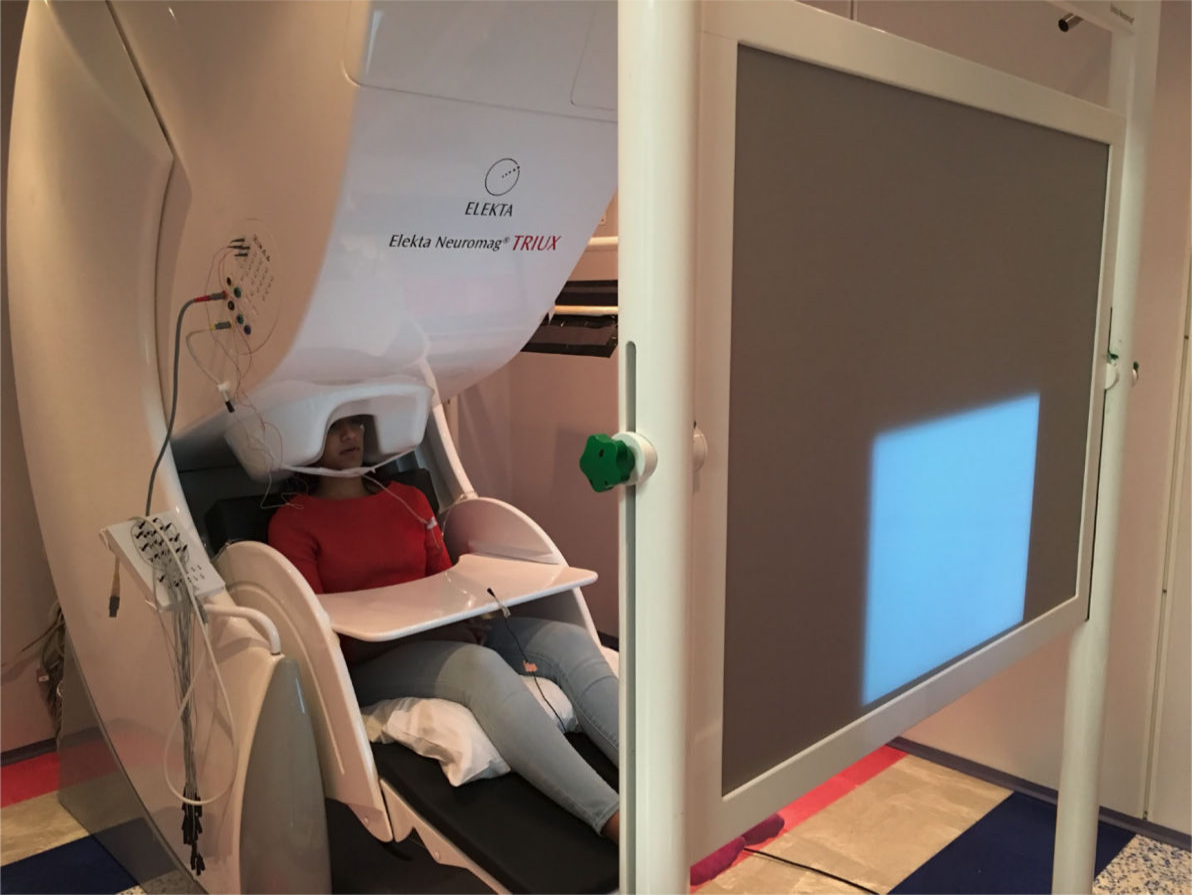
The MEG unit (Neuromag; MEGIN, LCC) with a subject. The MEG was kept at upright position with the subject sitting comfortably with their head inside the dewar (sensor cap). The screen was placed at about 90 cm distance from the subject which displayed the stimuli.

Two identical Triux Neuromag MEG devices (MEGIN, LCC) were used for neuromagnetic signal acquisition at Dell Children’s Medical Center, Austin, TX, USA and Cook Children’s Medical Center, Fort Worth, TX, USA (FIGURE 1). The MEG system has 306 channels with 102 magnetometer and 204 gradiometer sensors and is housed inside a magnetically shielded room (MSR). Data were recorded with a 4000Hz sampling rate with an online hardware filter of 0.03–1000Hz. Prior to MEG data acquisition, a subject head coordinate system based on three fiducial points was created using the FastTrak digitization system (Polhemus Ltd.). Five head-position-coils placed on the scalp were also digitized to facilitate head localization within the MEG helmet. Bipolar electrooculography (EOG) and electrocardiograph (ECG) sensors were used for recording the eye movement and cardiac signals, respectively. A standard microphone with a transducer situated outside the MSR was used to record the vocal responses from the subjects. A custom-built pressure sensor attached to an air bladder placed on the chin was used to record jaw movements. Both acoustic and jaw movement signals were provided to the MEG system Analog to Digital Converter (ADC) separately in real-time for digitization. Sensors were calibrated prior to data acquisition.

### D. PROTOCOL

**FIGURE 2.**
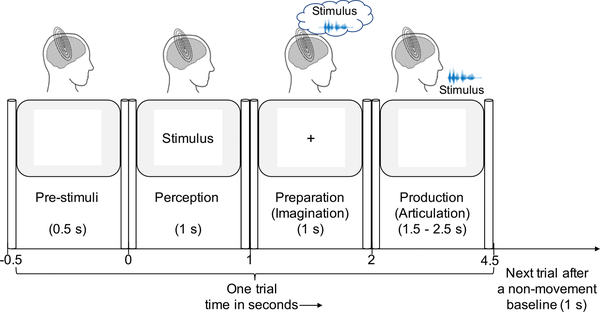
Experimental design of the time locked protocol. One trial consisted of 4 stages successively. The ‘0’ in the figure represents the stimulus onset. During the stage before stimulus onset (pre-stimuli) the screen was blank. After 0.5s of pre-stimuli stage, a sample stimulus out of the 5 stimuli was displayed on the screen that remained there for 1s. Then a fixation cross (‘+’) replaced the stimulus on the screen during the preparation/imagination stage. After 1s, the screen went blank and the subject overtly spoke the phrase during the production/articulation stage which lasted for 1.5–2.5s. There was a non-movement baseline of 1s before the start of the next trial during which the screen was blank as well.

The experiment was designed as a time-locked delayed overt reading task as shown in FIGURE 2. Each trial was time-locked to stimulus onset. Each trial started with a 1-second presentation of a stimulus phrase on the screen. This was followed by a delay period of 1 second where the stimulus was replaced by a fixation cross (+) heralding the subject to imagine and prepare for the articulation (production) of the previously shown phrase. The removal of the fixation (blank screen) cued the subject to articulate the previously shown stimulus at their natural speaking rate. Considering the difficulty in verifying the behavioral compliance of imagined speech production [62] we collected speech imagination and production data consecutively, within the same trial, where the timing of this paradigm constrained the subjects to imagining/preparing the same phrase which is expected to be articulated for the trial. The inter-stimulus interval was 5 seconds and the first 500ms prior to stimulus onset served as the baseline. The 5 phrases were displayed on the screen in pseudo-randomized order to avoid response suppression from repeated exposure [63], [64]. Each trial was repeated 100 times per phrase for each subject. The whole experiment lasted for about 45 minutes per subject.

### E. PREPROCESSING

The acquired signals were epoched into trials from −0.5 to 5s centered on stimulus onset. The MEG signals were low-pass filtered below 250Hz with a 4*^th^* order Butterworth filter and resampled to 1kHz. Line noise (60Hz) and harmonics were removed with a notch filter. Only gradiometer sensor data were considered for decoding due to their effectiveness in noise suppression. Sensors that showed a flat or overly noisy response were discarded, leaving 196-dimensional gradiometersensordataforanalysis.Throughvisualinspectionoftrials containing large artifacts, excessive eye blinks (checked from EOG data) or erroneous trials (subjects spoke before the cue) were removed. After preprocessing, a total of 2685 trials were retained out of 3500 recorded trials (7 subjects × 5 phrases × 100 trials). Although the average number of retained trials was 75 per phrase per subject, the data for one subject had only 63 valid trials for a phrase after preprocessing. Thus, for an unbiased comparison, we considered only the first 60 trials per phrase per subject for decoding.

## III. DECODING METHODS

The objective of this study was to identify the optimal MEG channels that are most critical for decoding speech imagination and production. For this, we implemented an SVM classifier based forward selection algorithm which iteratively selects an optimal set of sensors until the decoding performance converges. Further, we explored a stacked-autoencoder-based dimension reduction strategy to shrink the high dimension of MEG data onto the dimension of the optimal-set for verifying the improvement in decoding performance. Each of these two methods is briefly discussed below along with feature and classifier selection. The decoding task was a subject-dependent 5-class classification of the neuromagnetic (MEG) signals collected during the imagination and the production stage corresponding to the 5 phrases. We separated the signals of the imagination stage and the production stage into two separate tasks and performed the decoding on each task separately.

The core idea of this investigation was to use a forward selection algorithm to choose a smaller set of number of MEG sensors that have a similar performance level with that using all sensors. Speech decoding performances (accuracies) using data from the selected sensors were compared with these from the whole sensors using statistical analysis. If the speech decoding performance from the selected sensors was not significantly lower than these with the whole sensor set, we think the optimal set was found. We also performed additional analysis using a latest feature reduction technique, autoencoder, to confirm the speech performance using the forward selection algorithm.

### A. FEATURE EXTRACTION

Post preprocessing, the signals were decomposed with Daubechies-4 (db4) wavelet with 7-level decomposition. The signals were of 1kHz sampling frequency, from which the first two detail coefficients (d_1_ and d_2_) of frequencies 250 – 500Hz and 125–250Hz were discarded as noise. The detail coefficients (d_3–7_) and the approximation coefficient (a_7_) were reconstructed back to the 1000Hz sampling frequency representing all the neural oscillations such as high-gamma (61 – 125Hz), gamma (31 – 59Hz), beta (16 – 30Hz), alpha (8 – 16Hz), theta (4 – 8Hz), and delta (0.03 – 4Hz) respectively. Considering the effectiveness of db-4 wavelet in increased SNR and decoding performance in our prior works [12], [46], [65] and its use in other decoding studies [25], we implemented this denoising and decomposing step prior to decoding. The use of wavelets to generate distinct MEG brainwaves has been shown previously [67]–[69].

The root mean square (RMS) value across the 1s time segments of interest was extracted from the decomposed signals for each of the frequency bands (high-gamma, gamma, beta, alpha, theta, and delta). This resulted in an 1176-dimensional feature vector (196 sensors × 6 frequencies) for one trial per segment. The use of the RMS features was also motivated by our prior work and other decoding studies [20], [25], [46], [65]. Previously, we had observed that among all the statistical features (mean, median, standard, deviation, quartiles, tertiles), RMS was the only feature, which was significantly different across the 5 phrases (1-way ANOVA, posthoc Tukey test: *p <* 0.05). Since we considered only 60 trials per phrase per subject for decoding, the input feature matrix corresponding to each phrase was 1176 × 60. The motivation for this study to reduce the number of sensors and thereby the dimension can be appreciated from the dimension of the feature matrix where the feature size is much higher than the sample size (1176 *>* 300 (60 trials × 5 phrases)).

### B. CLASSIFIER

Considering the high-dimensionality of the input features, we chose a support vector machine (SVM) classifier for the decoding task. In contrast to neural networks, SVMs depend only on the data points near the separation boundary (support vectors), hence, they can be preferable for smaller datasets with large feature dimensions [69]. The classification was performed with a 5-fold cross-validation (CV) strategy using a 2*^nd^* order polynomial kernel. The choice of this 2*^nd^* order kernel was motivated from our prior work [45], [70] which found that this kernel performs optimally compared to other kernels (radial basis function (RBF), sigmoid, 3*^rd^* and 4*^th^* order polynomial) for this MEG data. C parameter tells the SVM optimization how much is needed to avoid misclassifying each training example. Tuning of this C parameter was performed for each classification task but did not provide any significant improvement and was fixed to unity. The input to the classifier was the 1176-dimensional RMS feature vectors with 60 trials per phrase. The 5-fold CV strategy divided the data into 5 equal folds (12 trials per phrase per fold) and used 4 folds (48 trials per phrase) for training and 1-fold (12 trials per phrase) for validation/test. Five subsequent training and validations were performed such that each fold was validated once. The average accuracy across the 5 folds was taken as the final performance. The feature dimension of 1176 is when all sensors were used to find the baseline accuracy.

### C. DECODING WITH ALL, LEFT, AND RIGHT SENSORS

As a baseline, first, we trained the SVM with the features extracted from all the gradiometer sensors to classify the phrases during imagination and articulation. The input feature dimension for All” was 1176. Next, for understanding the laterality in neural speech decoding we only took the sensors from the left hemisphere in MEG coordinates and trained the SVM classifier and repeated for right hemisphere sensors. The input feature dimension for “Left” and “Right” sensors decoding was 588. Same 5-fold cross-validation strategy and hyperparameters (e.g. C = 1, Kernel = 2*^nd^* order polynomial, etc.) as mentioned in the previous sections were used for the training of the classifier.

### D. FORWARD SELECTION ALGORITHM FOR OPTIMALSENSOR SET SELECTION

**FIGURE 3.**
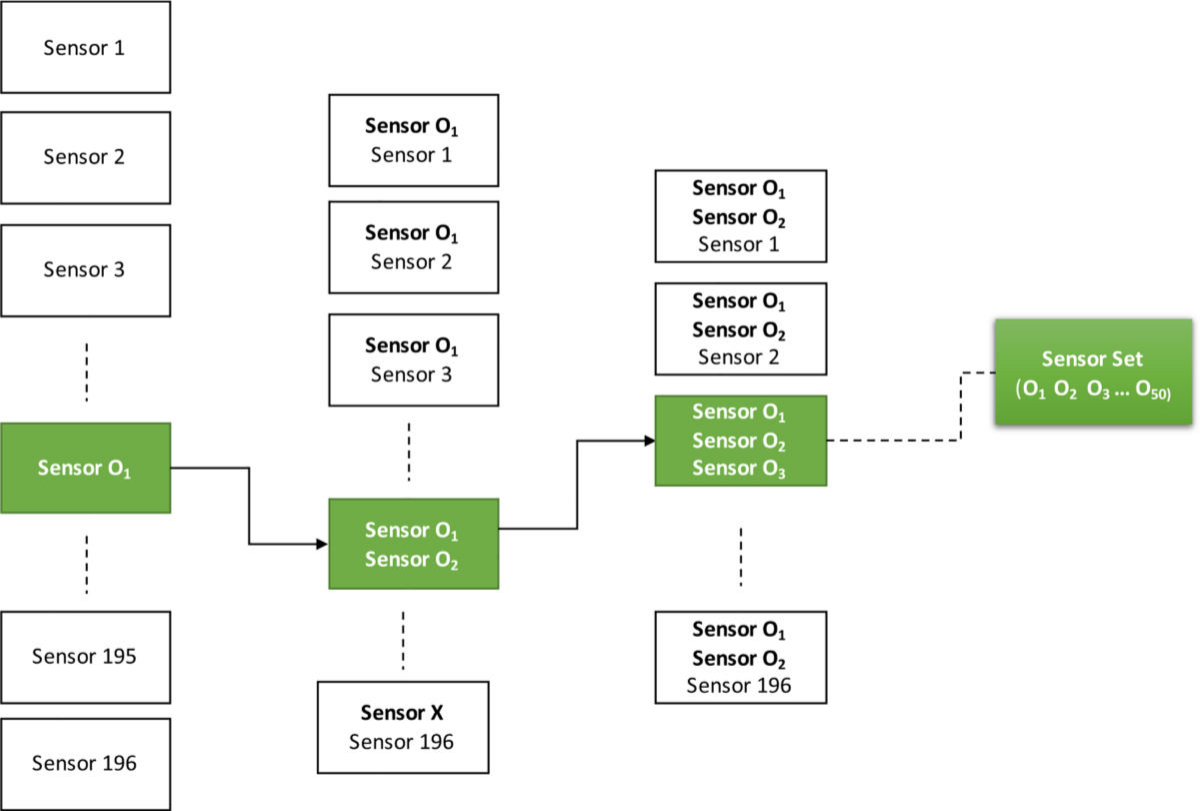
Forward sensor selection algorithm: This step-wise selection algorithm selects the most optimal set of sensors starting from 1 to 50, one by one in each step. First, the algorithm selects the first optimal sensor (O_1_) which results in best CV accuracy when the SVM was trained with each sensor as input for 196 times (total number of sensors = 196). Then with O_1_ fixed the SVM was trained for each pair of sensors to find the pair (O_1_ and O_2_) giving the best CV accuracy. Then with O_1_ and O_2_ fixed, it was trained with all possible sets of 3 sensors and so on until the accuracy converged with the maximum bound of up to a set of 50 optimal sensors. Please note the O_1_, O_2_...can be any sensor from 1 to 196 but all are different from each other.

The forward selection algorithm was designed in a step-wise manner (FIGURE 3) such that it adds one sensor to the optimal set in each step starting from the sensor giving the best CV classification accuracy in decoding the 5 phrases. In the first step of the forward-selection algorithm, the SVM classifier was first trained and cross-validated with features from each sensor one at a time as the input, i.e., using a 6dimensional feature vector (1 sensor x 6 frequencies). The sensor giving the best CV accuracy (e.g., O_1_) was added to the optimal set. Keeping the first optimal sensor (O_1_) fixed, the second step evaluated the performance of the remaining sensors when paired with O_1_, i.e., a 12-dimensional feature vector. The sensor that improved performance the most (O_2_) was then added to the optimal set. This process was repeated in an iterative manner where at each step, a new sensor’s efficacy was evaluated based on its ability to improve the classification performance of the previously selected group of sensors. This step-wise sensor selection process continued until the optimal set had 50 sensors (O_1_, O_2_, …O_49_, O_50_). We tested the forward selection algorithm up to 50 optimal sensors as in our previous work on speech decoding with principal component analysis (PCA) components of MEG signals [11], we found that the decoding performance saturated after 50 principal components. The forward selection algorithm was trained for the imagination and the production stage separately. Then the optimal set from the pool of 50 sensors was determined until saturation in average accuracy (across 7 subjects) was achieved.

### E. AUTOENCODER FOR DIMENSION REDUCTION

**FIGURE 4.**
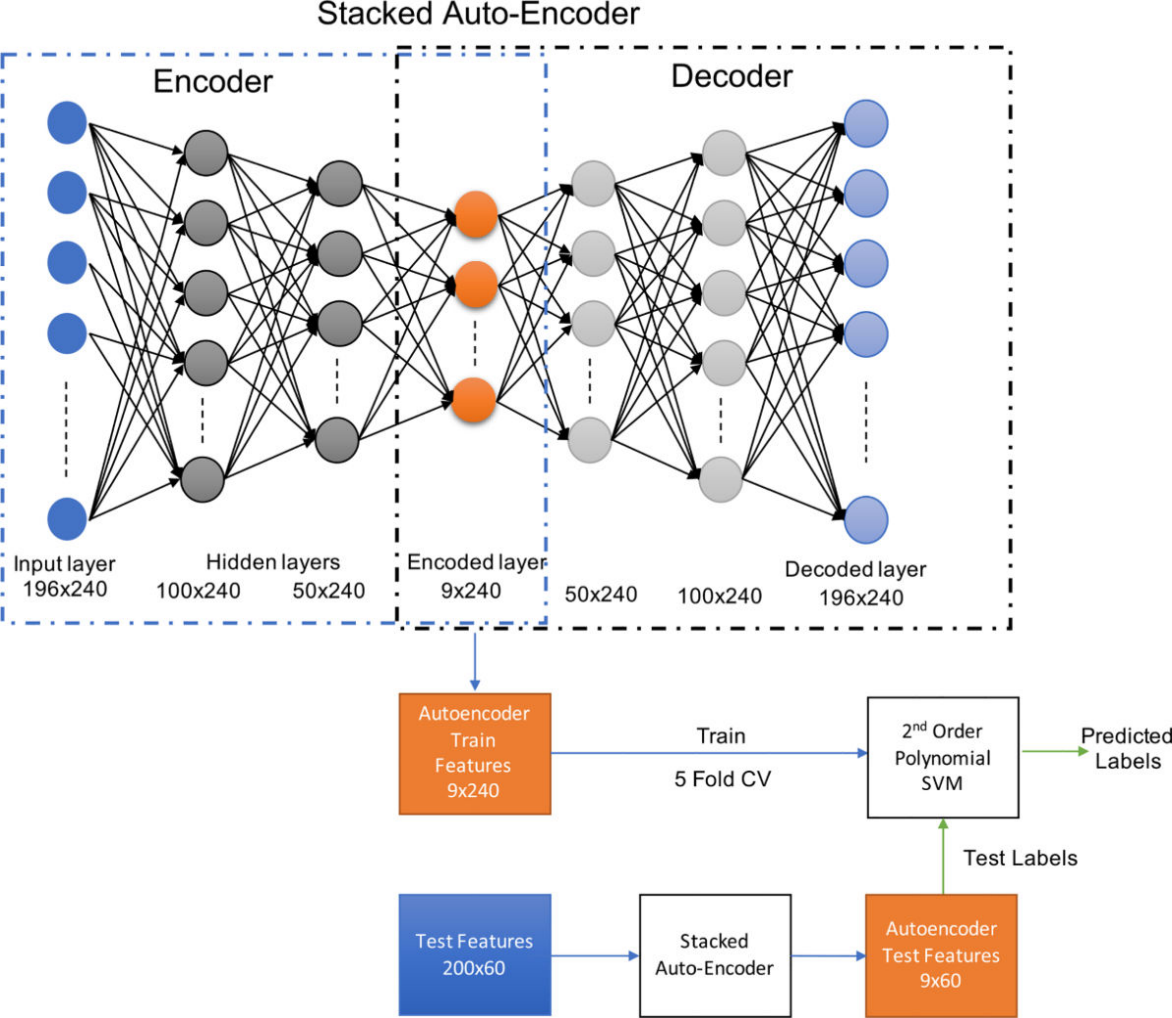
The architecture of the stacked-sparse autoencoder (AE) for dimension reduction. For training, the input to the AE was the 1176-dimensional (196 sensors × 6 brainwaves) RMS features of 240 training trials (48 trials per phrase × 5 phrases). The encoder-decoder architecture of this AE was designed such that the encoder first finds the most significant features in 54 dimensions (equivalent to feature dimension of 9 sensors: 9 sensors × 6 brainwaves) such that the decoder can generate the original input feature of 1176 dimension (equivalent to feature dimension of 196 sensors) from the low-dimensional (54) embedded features. Both encoder and decoder have 2 hidden layers of a successively reduced dimension of 600 and 300. After unsupervised pre-training the autoencoder and fine-tuning the stacked autoencoder with the training labels via a SoftMax network, the final 54-dimensional embedded feature was trained with a 2^*nd*^ order polynomial SVM with a 5-fold CV. Then the 1176 dimensional RMS features of 60 test trials (12 per phrase) were fed to the AE (without the labels) to generate the 54 dimensional test AE features, which were tested with the trained SVM model to predict the labels of thetest phrases.

We used a stacked-sparse autoencoder for dimension reduction to encode the high dimensional input features (1176-dimensional) onto a low-dimensional embedded layer, which were then used to train the SVM decoder (FIGURE 4). We stacked 3 pre-trained autoencoders with hidden layer nodes of 600 (equivalent to feature size of 100 sensors), 300 (equivalent to feature size of 50 sensors), and 54 (equivalent to feature size of 9 sensors) to form the stacked-autoencoder. Instead of directly embedding the large dimensional input feature (dimension = 1176) to a low dimensional embedded vector (dimension = 54) with one autoencoder, we chose to introduce two more autoencoders with a subsequent 50% less number of nodes than the previous layer to represent complex regions for managing non-linearity in the data. We chose the dimension of the 3*^rd^* hidden layer (embedded layer) based on the performance of the forward selection algorithm convergence. We adopted a greedy training strategy to first rain each of the autoencoders one by one in an unsupervised manner and then the autoencoders were stacked and fine-tuned in a supervised fashion with the corresponding labels. Each of the three autoencoders used was a 2-layer shallow neural network with the hidden layer as the encoding layer that embedded the input of the network and the decoding layer with the same size as the input layer to reconstruct (decode) the input. For example, the first autoencoder had an input layer dimension of 1176 (same as the input feature dimension), the hidden layer dimension of 600, and the output layer dimension was 1176, the same as the input layer. During the pre-training of the first autoencoder, the 1176-dimensional input was embedded onto the hidden layer of 600 nodes by removing the redundancy, from which the original input was trained to be decoded at the output layer. Similarly, for the 2*^nd^* autoencoder, the 1*^st^* hidden layer of 600 nodes became the input layer which is encoded to a hidden layer of 300 nodes. Finally, for the 3*^rd^* autoencoder, the information from the 300 nodes were encoded within 54 nodes.

We pre-trained each autoencoder with a scaled conjugate gradient (SCG) optimizer via backpropagation. Logistic sigmoid functions (logsig) were used for both encoder and decoder transfer functions. L_2_ norm on the weights and sparsity constraint on activations were used as regularization. The loss function was mean squared error adjusted for sparse autoencoders (MSEsparse), i.e., mean square error loss with added L_2_ norm regularization and sparsity regularization [71]. We used the default Kullback-Leibler divergence as the sparsity regularizer which attempts to enforce a constraint on the sparsity of the output from the hidden layer by adding a regularization term that takes a large value when the average activation value of a neuron and its desired value are not close [71]. Values for the coefficients of L_2_ weight regularizer, sparsity regularizer, and desired proportion for sparsity was decided based on grid search tuning for each encoder and each subject separately. Commonly, the L_2_ regularization factors for different subjects were found to be optimal within 0.001 – 0.004, the sparsity regularization factor was 4, and the sparsity proportion values were within 0.2 for the first AE, 0.15 for the second AE, and 0.10 for the third AE. All the encoders were trained for a maximum number of 400 epochs. After pre-training the three autoencoders, they were stacked and fine-tuned with a SoftMax layer with respective labels.

## IV. RESULTS

### A. DECODING PERFORMANCE WITH ALL, LEFT, AND RIGHT SENSORS

**FIGURE 5.**
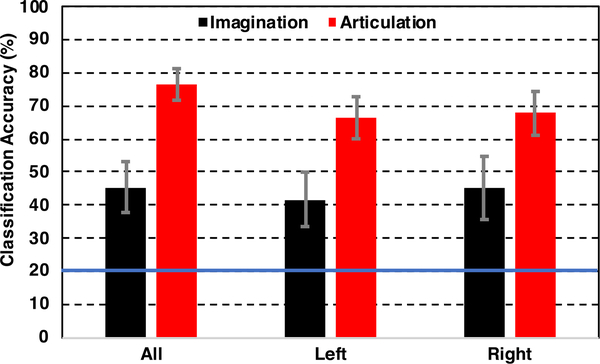
Comparison of decoding accuracies during imagination and articulation averaged across 7 subjects with *All*: Using all 196 sensors, *Left*: Using sensors on the left hemisphere, *Right*: Using Sensors on the right hemisphere. The blue solid line on 20% represents the chance level accuracy. Error bars indicate standard error (SE) across 7 subjects.

FIGURE 5 shows the results obtained when decoding with all sensors as a baseline, left hemisphere only, and right hemisphere only sensors. During imagination, the average accuracy with only the left sensors was 41.63% ± SE: 8.11%, and with only the right sensors, it was 45.16% ± SE: 9.39%. This difference was not significant (2-tail paired *t*-test, *p* = 0.48). Similarly, for articulation, the average classification accuracy with left-only sensors was 66.62% ± SE: 6.30%, and with right-only sensors, the mean accuracy was 67.83% ± SE: 6.60% across 7 subjects. A 2-tail paired *t*-test comparison of left and right lateral sensors for articulation decoding similarly demonstrated no statistically significant difference (*p* = 0.46). There was about a 10% decrease in mean performance from using all sensors to using only left or right hemisphere sensors, which was statistically significant (1-tail paired *t*-test, *p* = 0.004 for all v. left and *p* = 0.007 for all v. right). For imagination decoding, this decrease was minimal and not significantly lower than using all the sensors (1-tail paired *t*-test, *p* = 0.20 for all v. left and *p* = 0.44 for all v. right). The performance with both left- and right only sensors was also significantly higher than the theoretical chance level accuracy of 20% (1-tail t-test, *p <* 0.05).

### B. OPTIMAL SENSOR SET

**FIGURE 6.**
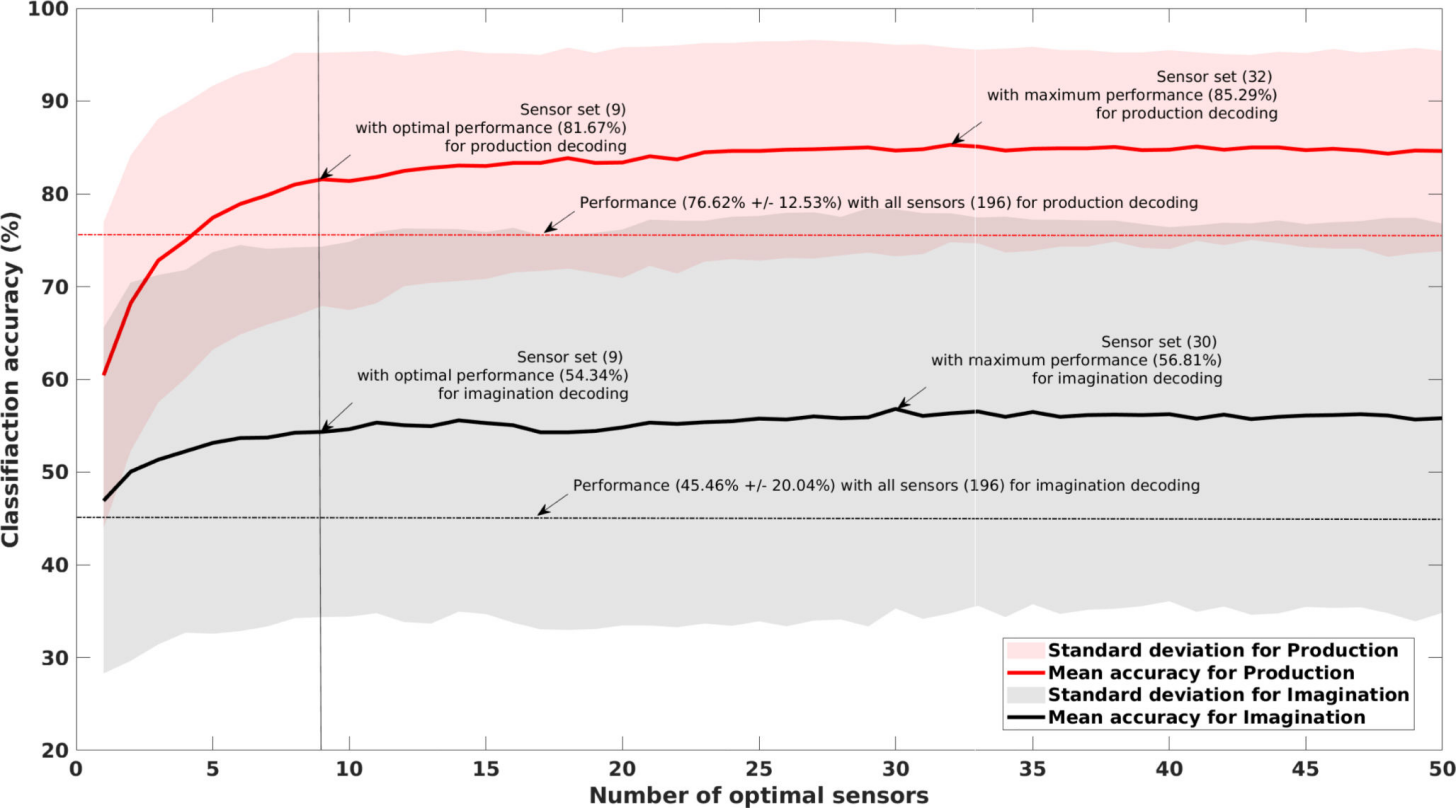
Average decoding performance across 7 subjects with the increasing number of optimal sensors for imagination and production. The solid red line corresponds to the classification accuracies for decoding spoken phrases as the number of optimal sensor increases and the corresponding standard deviation is plotted as a pink transparent shadow. The dotted red line represents the accuracy obtained by considering all sensors. Similarly, the black solid line is for imagined speech decoding accuracy with the gray shadow indicating the standard deviation and dotted black line for imagination decoding with all sensors. The number of sensors with optimal accuracy and the best accuracy for both imagined and spoken phrase decoding are shown with arrows.

We computed the performance of each optimal set starting from the best sensor through the 50 optimal sensors based on the forward selection algorithm. The results are shown in FIGURE 6. The decoding performances using all 196 sensors (feature dimension = 1176), calculated for both imagination and articulation, are shown as the black and red dotted lines on the plot respectively. For imagination, the 5-class average classification accuracy across 7 subjects with the SVM decoder was 45.46% ± 20.04% and for articulation, it was 76.62% ± 12.53%. The performances for articulation decoding were statistically significantly higher than imagination and the performances for both imagination and articulation were significantly higher than chance level (theoretically 20% for a 5– class classification) (1-tail t-test, *p <* 0.05).

Accuracy for both imagination and articulation decoding increased with the increase in the optimal number of sensors as can be seen in FIGURE 6 from the line plots (red solid line for articulation and black solid line for imagination). The standard deviations are plotted in shadows with the respective colors. For production (solid red line) a decrease in accuracy can be observed when the optimal number of sensors increased from 9 to 10 leading to algorithm convergence. However, we continued to evaluate the performance by adding more optimal sensors until 50 to observe the pattern. A plateau was observed after the 9*^th^* optimal sensor both for imagination and articulation. For articulation, with 9 optimal sensors, the average decoding accuracy was 81.67% ± 13.75% and for imagination, it was 54.34% ± 19.38%. On average, an increment of 9% for imagination and 5% for articulation was observed with only 9 optimal sensors compared to all 196 sensors. The increment was statistically was with 32 sensors during articulation (85.29% ± 10.53%) significant (1-tail paired t-test, *p* = 0.03 for articulation and with 30 sensors for imagination (56.81% ± 20.74%). and *p* = 0.001 for imagination). The maximum accuracy As the gain in performance is somewhat minor relative to the cost increase associated with the 21 additional sensors, we considered the 9 sensors as the optimal set.

### C. SENSOR LEVEL SPATIAL PATTERNS

**FIGURE 7.**
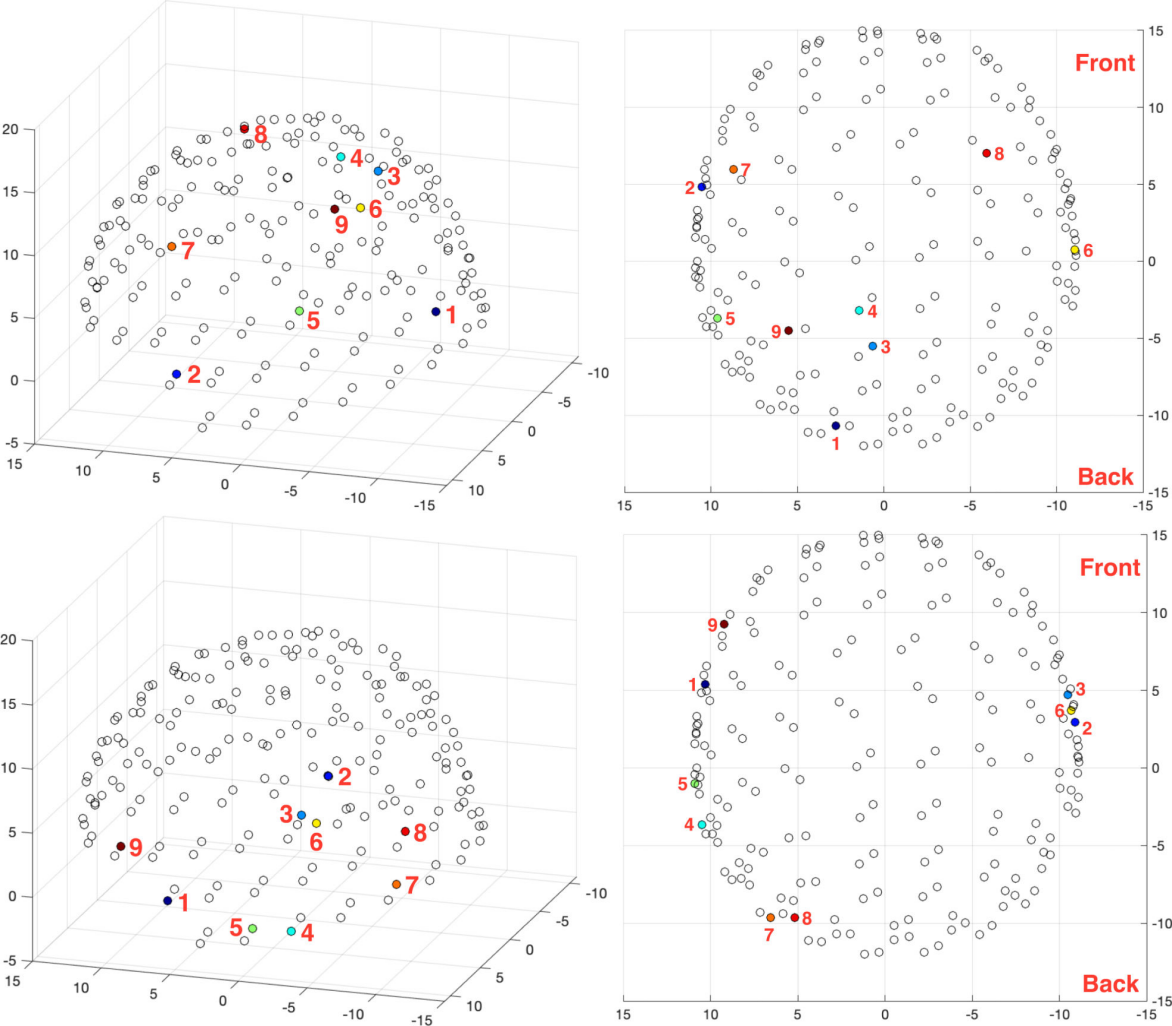
Tracking the locations of the optimal sensors in the 3D (left) and 2D-axial (right) sensor location space chosen by the forward selection algorithm for imagination (top) and production (bottom). Axial plots are shown to infer the lateral side (left/right) information. Rank is shown next to the sensor. The axes coordinates are in cm.

The locations of the optimal 9 sensors for one subject both for imagination and articulation are shown in FIGURE 7. The optimal sensors are shown in colors with the ranking shown in the plot. The best sensor, i.e., the sensor resulting in the best accuracy among all 196 sensors in the first step of the forward selection algorithm is shown in dark blue color. The 9*^th^* optimal sensor is represented with a dark red color. Following the change in color with corresponding ranking numbers, the ranking of the optimal set can be visualized. From the axial plots, the laterality (left/right) of the sensors can be inferred. Interestingly, the optimal sensors were mostly near the temporal regions of the brain for articulation decoding. However, the location of optimal sensors for speech imagination decoding was considerably different compared to articulation decoding with additional preference towards sensors near the occipital region.

**FIGURE 8.**
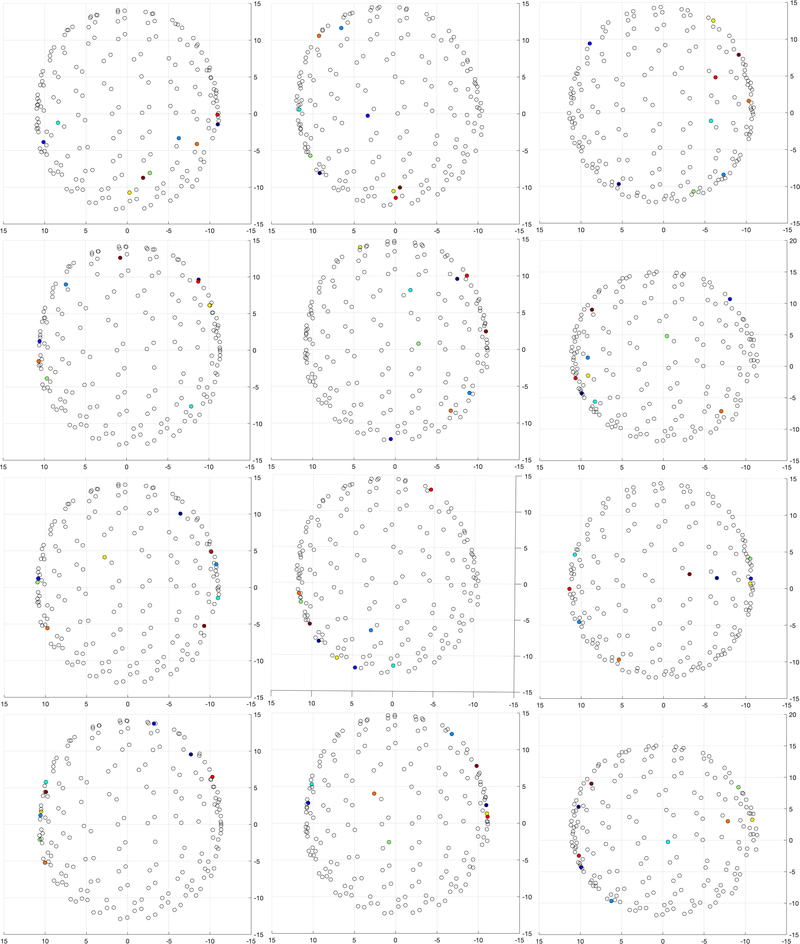
Inter-subject variability in the location of optimal sensors shown for 6 subjects for speech imagination (top two rows) and production (bottom two rows) decoding. The axial view of the sensor map is shown. The axes coordinates are in cm. The sensor locations are based on individual subject’s head space.

The intersubject variability in the location of optimal sensors can be seen in FIGURE 8. Although there is some expected variation across subjects [59], the locations in FIGURE 8 reflect a somewhat consistent inclusion of temporal region sensors for articulation and occipital region sensors for imagination. Sensors both at the left and right hemispheres were included in the optimal set in contrast to the traditional understanding of the left hemisphere dominance of the brain for speech production [72]. The bilateral distributions of optimal sensors were prominent for both imagination and articulation decoding across all subjects (FIGURE 7 and 8).

**FIGURE 9.**
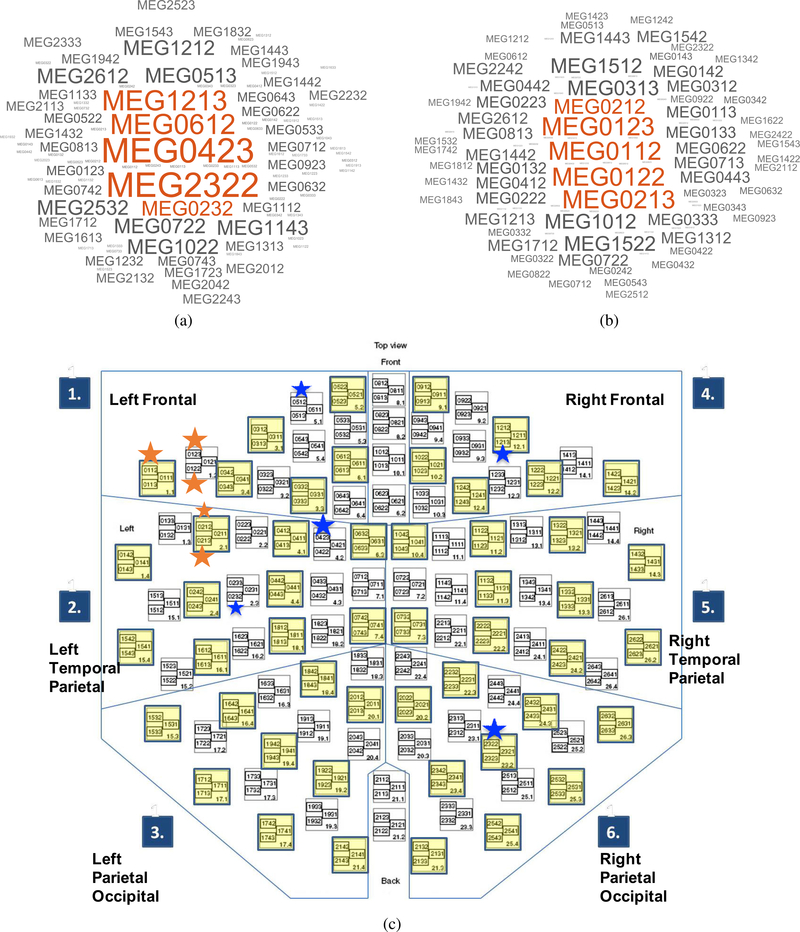
Statistical frequency (Mode) plot of the common sensors across 7 subjects during imagination (a) and articulation (b). a higher size of the text represents higher mode. The sensors with higher modes are also represented in orange color and at the center of the sensor-name cluster. For example, for the case of imagination decoding, sensor MEG2322 was common across all the 7 subjects. The location of the sensor can be inferred from the flattened sensor map with sensor names (c). Orange and blue colored stars (for articulation and imagination respectively) have been put near the index of the most common sensors for reference. Sensor layout image modified with permission, from user manual “TriuxTM neo Instruction for Use, 2020 (MEGIN, LCC).

FIGURE 9 provides the cluster of common sensor-names occurring in the top 50 optimal set across all subjects. Even though there was large variability in the top 9 optimal sensors, there were common sensors across the 7 subjects in a larger pool of optimal sensors (50) based on a statistical frequency (mode) analysis which identified the sensors that were often repeated across subjects. Higher font size indicates a higher mode. The most common sensors are represented in orange color at the center. For imagination, the sensors MEG2322 (right parietal-occipital) and MEG0432 (left temporal-parietal,) were common across all 7 subjects in the top 50 optimal sensors. The sensors MEG1213 (right frontal) and MEG0612 (left-frontal) were common to 6 subjects and so on. The results of the mode analysis on sensors chosen in the optimal set for articulation decoding was very intriguing. All the sensors shown in the center with orange color that are most common across 5 – 6 subjects were positioned near the fronto-temporal region which typically covers Broca’s area.

### D. DIMENSION REDUCTION WITH AUTOENCODER

**FIGURE 10.**
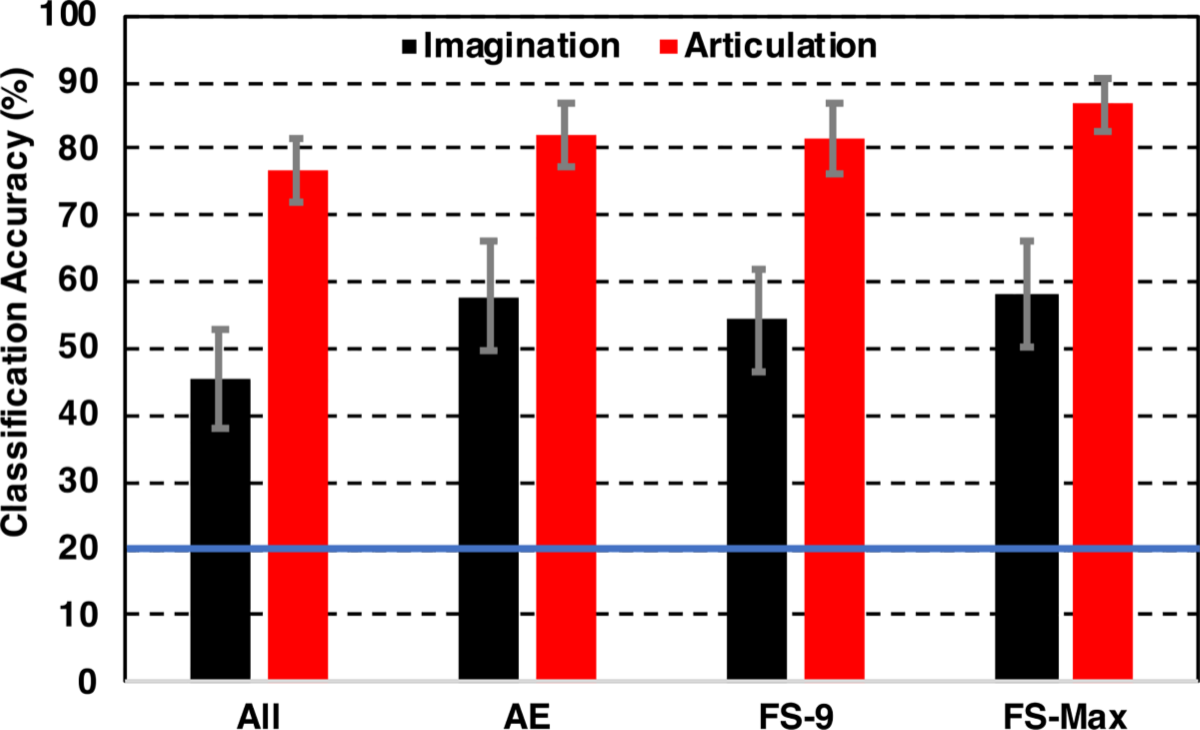
Comparison of decoding accuracies during imagination and articulation averaged across 7 subjects with *All*: using all 196 sensors, *AE*: after dimension reduction with autoencoder. *FS-9*: with 9 optimal sensors found with forward selection. *FS-Max*: with optimal sensors resulting in maximum average accuracy (30 for imagination and 32 for articulation; see figure 6) across 7 subjects after forward selection algorithm. The blue solid line on 20% represents the chance level accuracy. Error bars indicate standard error (SE) across 7 subjects.

After reducing the dimension from 1176 (of 196 sensors) to 54 (equivalent to 9 sensors) with the stacked-sparse autoencoder, the decoding performance increased by about 6% for articulation and 12% for imagination decoding (FIGURE 10). The average CV accuracies across 7 subjects, obtained with the SVM decoder with the autoencoder features with reduced dimension, were 57.92% ± *SE* : 8.07% for imagination and 82.08% ± *SE* : 4.96% for articulation. These accuracies were similar to those obtained with 9 optimal sensors. Also, this increment in mean performance with AE reduced features that from using all features (from 196 sensors) was statistically significant (1-tail paired t-test, *p* = 0.0005 for imagination and *p* = 0.0003 for articulation). Reducing the dimension of the third hidden layer with steps of 6 below 54 resulted in almost similar but lower accuracy (about 0.4% decrease for the first 2 steps) and then significantly decreased.

**FIGURE 11.**
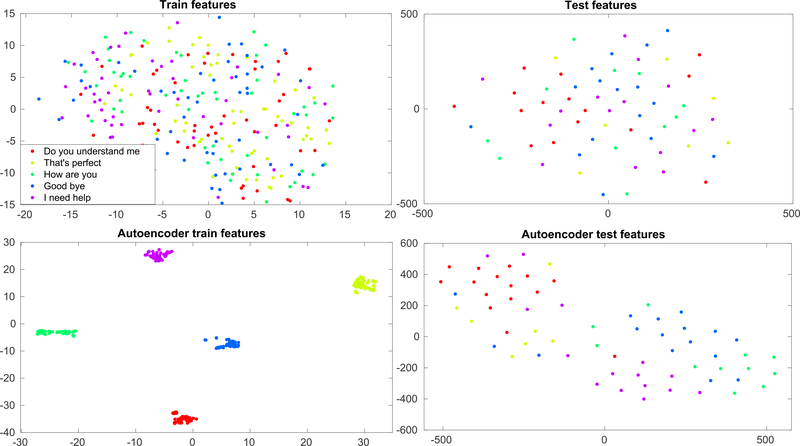
t-SNE plots of the train and the test features of a subject for articulation before and after dimension reduction with autoencoders. Each color represents a phrase (class). Each dot in train and test features (shown on top of the plot) is of dimension 1176 and after dimension reduction with AE (shown in the bottom of the plot), each sample (dot) is of dimension 54 obtained from the embedded layer of the stacked-sparse-AE.

FIGURE 11 shows the dimension reduction performance of the autoencoder from the feature distribution (t-stochastic neighbourhood estimation, t-SNE) plot [73]). The figure shows the distribution of train and test features of a subject during articulation, before and after being run through the autoencoder. Each dot in the plot represents a sample/trial and each color represents a class/phrase. The top two plots are samples with 1176-dimensional features of train and test respectively and the bottom 2 plots are the distribution of samples with reduced dimensions (54) via autoencoder. The samples are mixed in the original feature space and thus it is difficult for a decoder to find a boundary between different classes. However, with the reduced dimension the samples corresponding to different classes are clustered making the task of the decoder easy and thereby resulting in better performance.

## V. DISCUSSION

### A. EFFICACY OF THE SENSOR SELECTION STRATEGY

The observation that 9 optimal sensors outperform the original 196 sensor system (FIGURE 6) demonstrates the promise of a reduced number of sensors for neural decoding. We tested the forward selection algorithm up to 50 optimal sensors and found that the decoding performance saturated after 9 principal components. We chose nine to be our optimal set of sensors as the performance plateaued after 9 sensors. Although the maximum accuracy was achieved with about 30 optimal sensors, the increase in accuracy from 9 sensors to 30 sensors was only 2 – 4%, whereas the additional cost of those extra 21 sensors could be very high. In addition to being computationally efficient, a smaller set of sensors can reduce the cost of a BCI system dramatically. With the new era of OPM-MEG developments [47], [50], establishing the optimal placement of a few sensors with improved decoding performance may have a substantial impact on the feasibility of speech-BCIs. Based on our results, future, low-cost OPM-based speech decoders could be developed with as few as 9 sensors at a low cost. After getting the optimal location of the sensors via the traditional MEG system, on-scalp OPM sensors can be placed on those locations on a lightweight helmet for speech-BCI applications. Although to optimally select the 9 sensors, one still has to start with all the sensors, however, that can be done with a one-off calibration test before acquiring subject-specific devices. Alternatively, the used forward selection algorithm can be implemented on multi-channel dense OPM-MEG arrays to select the location of few OPM sensors that could potentially result in optimal decoding performance and a customized sensor set that is specific to the patient. Verification of these assumptions is necessary with OPM measured data, which will be a next step of this study.

There are several challenges associated with managing high-dimensional data. In addition to the computational difficulties, increasing the dimension of data exponentially increases the volume of the hypothesis space. This leads to a sparsely populated space in which it is difficult to draw decision boundaries. To avoid these challenges, there are a number of approaches, that aim at reducing the dimensionality of data while minimizing the loss of relevant information. Dimension reduction can be divided into feature selection strategies (which attempt to identify an optimal subset of features) and feature transformation strategies (which attempt to learn a transformation to a lower-dimensional space that preserves the most relevant information) [56]. Although feature transformation strategies are more flexible, and thus sometimes capable of learning more powerful low-dimensional representations, they lack some of the computational advantages of feature selection strategies as they generally still require that all of the initial features be calculated to build the low-dimensional representation. This is particularly relevant to this topic as dimensionality reduction strategies such as autoencoders or PCA would still require data from all of the initial sensors, and would thus be unhelpful in reducing the bulk or cost of the BCI. Because of this, the main focus of this paper is the implementation of feature selection strategies for neural speech decoding. From a machine learning, speech decoding, and cost perspective, we chose the forward selection algorithm in this study with the objective of choosing the lowest number of sensors, which has not been investigated before in MEG studies.

The forward selection strategy implemented here takes the concatenation of information from all the six brainwave frequencies for extracting features from the sensors. However, the spatial-spectral dynamics of brain cognition are very complex and it might be possible that a certain combination of optimally located sensors and different brainwaves might result in a better performance than taking all brainwaves simultaneously. For example, previous decoding studies have provided the supremacy of theta band dominance in decoding while speaking various words [74] or higher decoding performance by beta band compared to alpha and theta in classifying two syllables with EEG [21] and high-gamma band for speech decoding with ECoG [15]. However, in a recent MEG study, we showed that the best decoding accuracy can be achieved still by combining all the brainwaves [70]. Hence, we chose to take the concatenation of all six brainwaves as the feature for individual sensors in the current study. Future studies will be conducted to find the best combination of sensors with individual or a selection of combined frequency bands.

### B. SPATIAL PATTERNS OF THE OPTIMAL SENSOR SET

We found the 9 optimal sensors for articulation decoding were mostly near the temporal regions of the brain (FIGURE 8-bottom two rows). The effective role of the temporal cortex for speech processing has been previously shown for both perception and production [75]–[77] suggesting the functional role of speech areas (Broca’s area, Wernicke’s area, etc.). Interestingly, we found the optimal sensors to be distributed across temporal areas of both hemispheres in contrast to the traditional understanding of speech areas to be located at the left hemisphere only [72]. However, it is possible that the sensors might be reflecting the neural activity of the auditory cortex which is present in both hemispheres and, due to the field spread effect of MEG, sensors near the auditory cortex were picked by the algorithm. This also suggests that speech perception mechanisms during articulation might be contributing to the decoding process. A source reconstruction-based strategy might provide a clearer view of the spatial organization of the brain for speech production that is driving the optimal sensor set selection toward which our future work is progressing.

Interestingly, some sensors near the occipital regions of the brain were consistently included in the top 9 optimal sensors set during imagination decoding (FIGURE 8-top two rows). It might be possible that the subjects were imagining the previous visual stimulus, or that (visual) task-related activity gets incorporated into the neural dynamics related to the specific task. It is also possible that the visuomotor coordination might be involved in speech imagination during oromotor tasks [78], [79]. It might be possible to interchange the imagination and production tasks in the protocol to counterbalance this effect of task-related effects in half of the subjects during group subject analysis. The variability in the locations of the optimal sensors across subjects can be seen in FIGURE 8. It is also interesting that the optimal sensors for imagination decoding were distributed across hemispheres, supporting the existence of bi-hemispheric networks underlying speech production.

The observation that the optimal sensors obtained for speech imagination and production decoding are different suggests that these two tasks are considerably distinct cognitive processes even with the same speech stimuli. The optimal sensors for spoken speech decoding were mostly concentrated around the temporal lobe whereas were distributed across the entire brain for imagination. The ultimate goal of a speech-BCI is imagined speech decoding such that it can be used for ALS or locked-in patients for communication assistance. It may also be valuable to understand the speech production decoding analysis as it might provide further insights into the neurophysiology of speech production. Thus, extreme care must be taken in selecting the location for sensors corresponding to these two tasks.

The finding with statistical mode analysis of optimal sensors (Figure 9) could be seen as a direct data-driven reproduction of previous studies [80]–[82] implicating Broca’s region in speech production. Although the variability in sensor groups across subjects is relatively high, the observation that sensors near Broca’s area are common in the optimal pool provides certain confidence that in the future a subject-independent decoding model can be developed with few OPM sensors.

The bilateral representation of speech production has been repeatedly shown in the neuroimaging literature. This suggests a dynamic interaction/connection between the hemispheres [83] involving the bilateral auditory and motor cortices, rather the traditional understanding of speech production to be largely dominated by the left hemisphere (for right-handed speakers). In current neural speech decoding studies with ECoG, subjects with an electrode grid placed on the left hemisphere are typically chosen as subjects of interest. This is justified as it is very difficult to come across epileptic patients with source localized to both hemispheres. While the decoding results alone are insufficient to delineate a specific mechanism for bilateral speech processing, our results suggest that data from both the hemispheres can be used to maximize a BCI’s proficiency. Although this study included only right-handed participants, a future study including an equal number of left- and right-handed participants may inform more about the degree to which this spatial decoding method reflects hemisphere dominance.

### C. EFFICACY OF AUTOENCODERS IN DIMENSIONREDUCTION

When developing any neural speech decoding algorithm it is essential to consider the data that can be acquired for training, which is typically in the lower order of magnitude while collecting from the target population. Data modalities used in BCIs are generally highly complex requiring high-dimensional feature representations. Thus, developing a good decoder using machine learning, in the low data regime of speech-BCIs, is a major challenge. For a good machine learning decoder to perform well in this high-dimensional small sample regime, intelligent techniques must be developed including artificial data synthesis for increasing sample size, proper regularization of the decoders to avoid overfitting, dimension reduction approaches to reduce the feature size, or feature selection approaches to select the most meaningful features from the whole feature pool. Here, we implemented both dimensionality reduction and sensor selection strategy to address this challenge.

Autoencoders (AE) are very effective deep learning tools for reducing the dimension of high dimensional data to lower-dimensional coded non-linear features [84]. The stacking of autoencoders to boost the performance of deep networks was first shown in [85]. AEs are logically very effective in sensor level MEG studies as MEG data is typically characterized by high-dimensionality due to a large number of channels. Autoencoders have previously been effectively applied for dimension reduction of MEG features [60], [61]. Here, we wanted to ensure that the optimal sensors selected with the forward selection strategy were at par with deep learning-based dimension reduction strategies such as the AE used here. The mean performance obtained with AE selected features was similar (*<* 1%) to the performance obtained with 9 optimal sensors. This provides further confidence that a few (9) optimal sensors can be selected using a forward selection algorithm that can be equally effective as using all the gradiometer sensors. Further, the efficacy of the autoencoder was reconfirmed with the t-SNE plots for visualization of feature distribution. Both training and testing samples were found to be clustered into five different groups corresponding to five different phrases after implementing autoencoder based dimension reduction. The clustering is more prominent in training samples as these are fine-tuned with corresponding labels while training the stacked autoencoder. Nevertheless, the relatively better clustering of autoencoder test features compared to the original test features can still be seen in the plot and the efficacy of autoencoders for dimension reduction can be inferred.

While developing a BCI, it is extremely difficult to collect a lot of training data from the subjects considering the fatigue that can be developed while doing a task for a long time. Specifically, for the ALS population, subjects become tired easily during the task, considering the higher amount of motor involvement to compensate for the paralyzed muscles [86]. Thus, developing deep learning-based decoders on high dimensional neural data with a small number of training samples is challenging. Hence, in this study, we used SVM which is partially unbiased to high-dimension. It might be true that with a higher sample size, after training the autoencoder, a deep network-based decoder could have produced better performance. However, for an unbiased comparison, we used the same SVM decoder after both forward selection algorithm and autoencoder based dimension reduction strategy.

### D. TOWARDS AN OPM-MEG-SPEECH-BCI

An ideal BCI should be accurate, mobile, real-time, and inexpensive. Although MEG signals might seem to be the most effective neural pathway for BCI applications, the current MEG machines are not suitable considering the high cost and immobility. Nevertheless, recent studies on OPMs [48]–[51] have provided confidence that the neuromagnetic activity can be effectively leveraged for practical BCI systems. In light of the findings reported here, it seems that with a few optimal OPM sensors it is possible to develop a fast speech-BCI for communication assistance. Also, the OPM-MEG-Speech-BCI could be low cost (due to fewer (9) sensors), movable (as OPMs can tolerate head motion), fast (because of direct decoding), and more accurate (with high decoding accuracy). A practical speech-BCI typically uses a closed-loop paradigm in which the prediction of decoding can be fed to the system as feedback to further improve the decoding efficacy. In this study, our experiment for MEG sensor selection is an open-loop decoding paradigm, because our focus is the comparison of the performances of individual sensors and their combinations. A closed-loop system with potentially human in the loop paradigm for real-time speech-BCI applications will be investigated in future studies [87]. In this study, we have only focused on MEG sensor selection for decoding but, in the future, we will focus on implementing a closed-loop system with potentially humans in the loop paradigm for real-time speech-BCI applications.

It is important to note that our results were based on MEG gradiometer sensors rather than OPM sensors. Although the reproduction of these results with OPM sensors is necessary there are reasons to be optimistic as the quality of OPMs is improving and signals, albeit slightly noisier, are believed to be similar to current MEG systems [48]. Moreover, recent studies have shown by using interference cancellation via a reference array, OPMs can generate high SNR signals significantly better than gradiometers [47]. Furthermore, gradiometers provide higher SNR over magnetometers and it has been recommended to use gradiometers over magnetometers for sensor level analysis [64], [88], [89]. Additionally, this study was performed as a closed-set classification task (5 phrases) and the sensors included in the optimal set might be different for an open-set task (any phrase) or a different set of phrases. Nevertheless, using such a forward selection algorithm to find the optimal set of sensors could theoretically be applied to any decoding research including EEG-based classification studies as well. Validating the location of the sensor selection with a test-retest paradigm on new data and expanding the vocabulary of phrases are the future goals of this research.

## VI. SUMMARY

In this study, we used a forward selection strategy with SVM decoders to find that with only 9 optimal sensors a significantly higher neural speech decoding accuracy can be achieved thereby showing promise for a future OPM-MEG-based low-cost speech-BCI. An autoencoder-based dimension reduction strategy was used to find the decoding accuracy which was similar to the accuracy obtained with the 9 optimal sensors, further reinforcing confidence in the chosen sensors. Considerable inter-subject variability was observed in the location of the optimal sensors, however, fronto-temporal sensors (overlying Broca’s area) were found to be common in the optimal set across subjects during articulation decoding. Sensors in the temporal region and parietal-occipital regions were selected in the optimal set for articulation and imagination decoding, respectively. The bilateral distribution of optimal sensors indicates bilateral neural activity during speech is prominent and necessary for decoding. This study only included healthy subjects’ data collected with a SQUID-MEG system, hence, further investigation on ALS subject’s data with OPM sensors is necessary to confirm the reliability of this strategy for developing the future OPM-MEG-speech-BCIs.
